# A health system assessment approach to analysis of political parties' health proposals, Portugal

**DOI:** 10.2471/BLT.24.291831

**Published:** 2024-08-20

**Authors:** Sara Machado, Ana Moura, Francisca Vargas Lopes, Diogo Marques, Luis Sa

**Affiliations:** a Department of Health Services, Policy and Practice, Brown University School of Public Health,121 S Main St, Providence, RI 02903, United States of America.; b Amsterdam, Kingdom of the Netherlands.; c Erasmus School for Health Policy and Management, Rotterdam, Kingdom of the Netherlands.; d Lisbon, Portugal.; e NIPE-Centre for Research in Economics and Management, Universidade do Minho, Braga, Portugal.

## Abstract

**Problem:**

Comparing health policy measures before elections and identifying potential gaps in the health policy debate can be challenging.

**Approach:**

We explored the use of the Health System Performance Assessment for Universal Health Coverage framework to analyse health policy proposals by classifying health policy measures outlined in political manifestos into four health system functions: governance, financing, resource generation and service delivery. As a case study, we analysed the political manifestos of all Portuguese parties with parliamentary representation ahead of the election in March 2024. We calculated the share of measures per health system function for individual political manifestos and identified potential gaps in the health policy debate. When required, we used additional classification criteria and local expertise on political and institutional knowledge.

**Local setting:**

A snap general election was announced in Portugal in November 2023, following an alleged corruption scandal, and political parties began publishing their manifestos on their websites in January 2024.

**Relevant changes:**

We identified and classified 350 health-related measures across the four functions: governance, 29.7% (104 measures); financing, 16.9% (59 measures); resource generation, 33.4% (117 measures); and service delivery, 20.0% (70 measures). These findings enabled characterization of the priorities of parties, facilitated cross-party comparisons and identified missing topics in the political debate.

**Lessons learnt:**

We show that the framework can be adapted to analyse political manifestos, providing a systematic method for comparing and synthesizing health policy proposals. We further demonstrate the potential for extending the framework’s applicability beyond health system performance assessment, opening new avenues for policy analysis.

## Résumé

**Problème:**

Comparer les mesures de santé publique en amont des élections et identifier les éventuelles lacunes dans les débats sur la politique sanitaire peut se révéler complexe.

**Approche:**

Nous avons observé comment le cadre d'évaluation de la performance des systèmes de santé pour une couverture sanitaire universelle était utilisé afin d'analyser les propositions en matière de santé publique. À cette fin, nous avons classé les mesures figurant dans les programmes politiques dans l'une des quatre fonctions des systèmes de santé: gouvernance, financement, mobilisation des ressources et prestation des services. En guise d'étude de cas, nous avons étudié les programmes politiques de tous les partis portugais représentés au Parlement avant les élections de mars 2024. Nous avons ensuite calculé, programme par programme, la part réservée aux mesures concernant chaque fonction des systèmes de santé, puis repéré les éventuelles lacunes dans les débats sur la politique sanitaire. Le cas échéant, nous avons appliqué des critères de classification supplémentaires et une expertise locale portant sur les connaissances politiques et institutionnelles.

**Environnement local:**

Le Portugal a annoncé l'organisation d'élections générales anticipées en novembre 2023 après un scandale de corruption présumée, et les partis politiques ont commencé à publier leurs programmes sur leur site Internet en janvier 2024.

**Changements significatifs:**

Nous avons répertorié et classé 350 mesures en lien avec la santé publique selon les quatre fonctions précitées: gouvernance, 29,7% (104 mesures); financement, 16,9% (59 mesures); mobilisation des ressources, 33,4% (117 mesures); et prestation des services, 20,0% (70 mesures). Ces résultats ont permis de définir les priorités des partis, de faciliter les comparaisons entre eux et d'identifier les thèmes absents des débats politiques.

**Leçons tirées:**

Nous apportons la preuve que le cadre peut être adapté en vue d'analyser les programmes politiques, offrant ainsi une méthode systématique pour comparer et synthétiser les propositions de santé publique. Nous révélons également les possibilités d'étendre le champ d'application de ce cadre au-delà de l'évaluation de la performance des systèmes de santé, ce qui ouvre de nouvelles perspectives dans le domaine de l'analyse politique.

## Resumen

**Situación:**

Comparar las medidas de política sanitaria antes de las elecciones e identificar posibles lagunas en el debate sobre políticas sanitarias puede ser todo un reto.

**Enfoque:**

Se exploró el uso del marco de Evaluación del rendimiento del sistema sanitario para la cobertura sanitaria universal con el fin de analizar las propuestas de política sanitaria mediante la clasificación de las medidas de política sanitaria descritas en los manifiestos políticos en cuatro funciones del sistema sanitario: gobernanza, financiación, generación de recursos y prestación de servicios. Como estudio de caso, se analizaron los manifiestos políticos de todos los partidos portugueses con representación parlamentaria ante las elecciones de marzo de 2024. Se calculó el porcentaje de medidas por función del sistema sanitario en los distintos manifiestos políticos y se identificaron posibles lagunas en el debate sobre política sanitaria. Cuando fue necesario, se recurrió a criterios de clasificación adicionales y a la experiencia local en materia de conocimientos políticos e institucionales.

**Marco regional:**

Se anunciaron elecciones generales anticipadas en Portugal en noviembre de 2023, tras un presunto escándalo de corrupción, y los partidos políticos comenzaron a publicar los manifiestos en sus sitios web en enero de 2024.

**Cambios importantes:**

Se identificaron y clasificaron 350 medidas relacionadas con la salud en las cuatro funciones: gobernanza, 29,7% (104 medidas); financiación, 16,9% (59 medidas); generación de recursos, 33,4% (117 medidas); y prestación de servicios, 20,0% (70 medidas). Estos resultados permitieron caracterizar las prioridades de los partidos, facilitaron las comparaciones entre partidos e identificaron temas ausentes en el debate político.

**Lecciones aprendidas:**

Se demuestra que el marco puede adaptarse para analizar manifiestos políticos, lo que proporciona un método sistemático para comparar y sintetizar propuestas de política sanitaria. Asimismo, se demuestra la posibilidad de ampliar la aplicabilidad del marco más allá de la evaluación del rendimiento de los sistemas sanitarios, abriendo nuevas vías para el análisis de políticas.

## ملخص

**المشكلة:**

قد يكون من الصعب مقارنة إجراءات السياسة الصحية قبل الانتخابات، وكشف الثغرات المحتملة في مناقشة السياسة الصحية.

**الأسلوب:**

لقد قمنا باستكشاف استخدام إطار العمل الخاص بتقييم أداء النظام الصحي من أجل التغطية الصحية الشاملة، وذلك بهدف تحليل مقترحات السياسة الصحية من خلال تصنيف إجراءات السياسة الصحية المُشار إليها في البيانات السياسية إلى أربع وظائف للنظام الصحي: الحوكمة، والتمويل، وتوليد الموارد، وتقديم الخدمات. وفي إحدى دراسات الحالة، قمنا بتحليل البيانات السياسية لجميع الأحزاب البرتغالية التي تتمتع بالتمثيل البرلماني قبل الانتخابات في مارس/آذار 2024. وقمنا بحساب حصة الإجراءات لكل وظيفة في النظام الصحي للبيانات السياسية الفردية، وحددنا الثغرات المحتملة في مناقشة السياسة الصحية. وعندما كان ذلك مطلوبًا، استخدمنا معايير تصنيف إضافية، وخبرة محلية في المعرفة السياسية والمؤسسية.

**المواقع المحلية:**

تم الإعلان عن انتخابات عامة مبكرة في البرتغال في نوفمبر/تشرين الثاني 2023، وذلك بعد فضيحة فساد مزعومة، وبدأت الأحزاب السياسية في نشر بياناتها على مواقعها الإلكترونية في يناير/كانون الثاني 2024.

**التغيّرات ذات الصلة:**

قمنا بتحديد وتصنيف 350 إجراءً متعلقًا بالصحة عبر الوظائف الأربعة: الحوكمة، %29.7 (104 إجراءً)؛ والتمويل %16.9 (59 إجراءً)؛ وتوليد الموارد، %33.4 (117 إجراءً)؛ وتقديم الخدمات %20.0 (70 إجراءً). وأتاحت هذه النتائج تمييز أولويات الأحزاب، وسهّلت من إجراء مقارنات بين الأحزاب، وحددت المواضيع المفقودة في النقاش السياسي.

**الدروس المستفادة:**

نحن نوضح أنه يمكن تكييف إطار العمل لتحليل البيانات السياسية، وتوفير طريقة منهجية لمقارنة وتقنين مقترحات السياسة الصحية. ونوضح كذلك إمكانية توسيع نطاق تطبيق إطار العمل إلى ما هو أبعد من تقييم أداء النظام الصحي، وفتح آفاق جديدة لتحليل السياسات.

## 摘要

**问题:**

在选举前比较卫生政策措施并确定卫生政策辩论中可能存在的差距具有一定挑战性。

**方法:**

通过将政治宣言中概述的卫生政策措施分为管理、筹资、资源生成和服务提供四大卫生系统职能，我们探索了全民健康覆盖卫生系统绩效评估框架在分析卫生政策提案过程中的使用情况。作为案例研究，我们分析了 2024 年 3 月选举前所有拥有议会席位的葡萄牙政党的政治宣言。我们计算了每个卫生系统职能相关措施在各个政治宣言中所占的比例，并确定了卫生政策辩论中可能存在的差距。必要时，我们还借鉴了其他分类标准和当地的政治和制度情况相关专家意见。

**当地状况:**

在出现所谓的腐败丑闻之后，葡萄牙于 2023 年 11 月突然宣布举行大选，各政党于 2024 年 1 月开始在其网站上发布竞选宣言。

**相关变化:**

我们根据四大职能确定了 350 项卫生相关措施并进行了分类，其中：管理，占 29.7%（104 项措施）；筹资，占 16.9%（59 项措施）；资源生成，占 33.4%（117 项措施）；以及服务提供，占 20.0%（70 项措施）。这些研究结果有助于我们确定各政党的工作重心，有利于跨政党进行比较，并查明政治辩论中遗漏的议题。

**经验教训:**

研究表明，该框架为比较和综合卫生政策提案提供一种系统的方法，可用于分析政治宣言。我们进一步证明了可以将该框架的适用性扩展到卫生系统绩效评估以外领域，为政策分析开辟了新的途径。

## Резюме

**Проблема:**

Сравнение мер политики в области здравоохранения перед выборами и выявление возможных пробелов в дебатах по вопросам политики в области здравоохранения может оказаться непростой задачей.

**Подход:**

Авторами было изучено использование системы оценки эффективности системы здравоохранения для обеспечения всеобщего охвата услугами здравоохранения в целях анализа предложений по политике в области здравоохранения путем классификации мер политики в области здравоохранения, изложенных в политических манифестах, по четырем функциям системы здравоохранения: управление, финансирование, генерирование ресурсов и предоставление услуг. В качестве примера приведен анализ политических манифестов всех португальских партий, представленных в парламенте, в преддверии выборов в марте 2024 года. По отдельным политическим манифестам была рассчитана доля мер по каждой функции системы здравоохранения и выявлены возможные пробелы в обсуждении политики здравоохранения. При необходимости использовались дополнительные критерии классификации и местный опыт в области политических и институциональных знаний.

**Местные условия:**

В ноябре 2023 года в Португалии были объявлены внеочередные всеобщие выборы после предполагаемого скандала по делу о коррупции, а в январе 2024 года политические партии начали публиковать свои манифесты на своих веб-сайтах.

**Осуществленные перемены:**

По четырем функциям было определено и классифицировано 350 показателей, связанных со здравоохранением: управление – 29,7% (104 показателя); финансирование – 16,9% (59 показателей); генерирование ресурсов – 33,4% (117 показателей); предоставление услуг – 20,0% (70 показателей). Полученные данные позволили охарактеризовать приоритеты партий, облегчили межпартийные сравнения и выявили недостающие темы в политических дебатах.

**Выводы:**

Показано, что эта система может быть адаптирована для анализа политических манифестов, что обеспечивает систематический метод сравнения и синтеза предложений по политике в области здравоохранения. Кроме того, демонстрируется возможность расширения применимости системы за пределы оценки эффективности системы здравоохранения, что открывает новые возможности для анализа политики.

## Introduction

Health and health care are key topics for voter decision. However, synthesizing the full range of policy proposals to compare the political parties’ vision for a country’s health system is challenging. We use the Portuguese snap general election of March 2024 as a case study to demonstrate how a structured health systems assessment approach can be used to synthesize political manifestos and inform the debate with data. To do so, we extended the use of the Health System Performance Assessment for Universal Health Care framework^1^ (hereafter referred to as the health systems assessment framework) to classify all health policy measures included in political party manifestos ahead of the election. 

The health systems assessment framework defines the four policy-modifiable functions of health systems: governance, financing, resource generation and service delivery.^1^ Typically, the framework is used to compare health systems and their performance or how particular policy changes affect health systems.^2^^,^^3^ We show how to use the framework to classify measures (policies) in manifestos according to the health systems assessment framework’s four health system functions. We also characterize each manifesto, as well as the overall health policy debate, according to the classification system.

## Local setting

A snap general election was announced in Portugal in November 2023, following an alleged corruption scandal (unrelated to health policy) and ending the ruling party’s parliamentary majority term two years early. Political parties began publishing their manifestos in January 2024. 

## Approach

We analysed the manifestos of the eight political parties holding parliamentary seats ahead of the election of March 2024. We retrieved the manifestos from parties’ websites in February 2024. These total approximately 80 pages on health policy.

Seven local health policy experts who are board members of APES, the Portuguese Health Economics Association^4^ (a non-partisan academic researcher-led body) identified and extracted all health policy measures from the manifesto text. To ensure data collection accuracy, each manifesto was independently analysed by two experts. 

Five of the seven above-mentioned experts (the authors of this study) then classified the measures according to the four functions outlined in the health systems assessment framework as follows. First, we independently classified each measure into the function that best captured its entry point into the health system – i.e. the function through which a measure is initially implemented or has its most immediate and direct impact (for example, the entry point of a change in copayments was classified as financing). We then reviewed the five independent classifications in expert consensus meetings during which a final classification was defined. We also defined additional guidelines whenever there were potentially multiple entry points. Two main issues led to multiple entry points. The first issue, text ambiguity, warranted knowledge of party rhetoric and political and institutional context to inform the classification. The second issue was inadequate fit into the health system assessment framework’s definitions. Specifically, some measures described either the provision of a new type of care or the increase in quantity of an existing type of care, as well as the required inputs. In such cases, despite reference to output (service delivery) in their proposals, we prioritized the reference to inputs (resource generation), as these are upstream of service delivery in a hypothetical production function of health care. This amounted to an expansion of the analytical framework defined in the health systems assessment framework.

Once all measures were classified according to health system functions, we computed the share of measures per function for each political manifesto. We then compared the resulting relative weight of each function across the manifestos.

## Relevant changes

Our analysis identified and classified 350 health-related measures from the manifestos. Once classified, these were distributed as follows: governance, 29.7% (104 measures); financing, 16.9% (59 measures); resource generation, 33.4% (117 measures); and service delivery, 20.0% (70 measures; Table 1). Table 2 showcases a sample of 35 measures.

**Table 1:** Number of health system measures in political manifestos, Portugal, 2024

Party	No. (%)			
	Governance	Financing	Resource generation	Service delivery
*Aliança Democrática* (50 measures)	13 (26.0)	10 (20.0)	11 (22.0)	16 (32.0)
*Bloco de Esquerda* (58 measures)	22 (37.9)	8 (13.8)	23 (39.7)	5 (8.6)
*CHEGA *(28 measures)	6 (21.4)	5 (17.9)	7 (25.0)	10 (35.7)
*Iniciativa Liberal *(24 measures)	6 (25.0)	5 (20.8)	7 (29.2)	6 (25.0)
*Livre *(45 measures)	14 (31.1)	8 (17.8)	19 (42.2)	4 (8.9)
*Partido Animais e Natureza *(68 measures)	16 (23.5)	16 (23.5)	24 (35.3)	12 (17.6)
*Partido Comunista Português *(24 measures)	10 (41.7)	3 (12.5)	10 (41.7)	1 (4.2)
*Partido Socialista *(53 measures)	17 (32.1)	4 (7.5)	16 (30.2)	16 (30.2)
**Total (350 measures)**	**104 (29.7)**	**59 (16.9)**	**117 (33.4)**	**70 (20.0)**

Note: Inconsistencies arise in some values due to rounding.

**Table 2:** Measures by health system function, extracted from political manifestos ahead of Portugal’s snap general election in March 2024

Function	Measure	Party
**Governance**		
Role of private providers	To reintroduce public–private partnerships in national health service hospitals	*Iniciativa Liberal*
	To outlaw public–private partnerships in national health service public hospitals as well as type C family health units (privately-owned primary care physician groups contracted to provide care for national health service patients)	*Bloco de Esquerda *
	To outlaw type C family health units	*Partido Comunista Português*
Electronic health records	To implement integrated health information systems: establishing a unified digital platform and universal electronic health records	*CHEGA*
Governance of public hospitals and the national health service board	To revise the national health service statutes, specifically by eliminating the role of national health service chief executive officer	*CHEGA*
	To restructure the national health service executive board, streamlining its organizational structure and redefining its functional responsibilities	*Aliança Democrática*
	To implement a top-down governance model for the national health service, with the national health service executive board at its core	*Partido Socialista*
	To promote democratic administration in national health service providers by introducing competitive recruitment for both hospital and primary care units’ chief executive officers, and allowing the workforce to elect their managers	*Partido Comunista Português*
Health system performance monitoring	To conduct regional and national audits of waiting time targets across the [national health service] hospital network	*Aliança Democrática*
	To strengthen health system performance monitoring by resorting to public reporting of key indicators	*Partido Socialista*
**Financing**		
Pay-for-performance		
Primary care	To foster the rollout of type B family health units (national health system-owned primary care physician groups contracted using pay-for-performance to provide care for national health service patients)	*Iniciativa Liberal*
	To reward the health workforce based on the quality of treatment and outcomes instead of activity-based payments	*Partido Animais e Natureza*
Hospital care	To offer additional monetary incentives (within the *Sistema Integrado de Gestão de Inscritos para Cirurgia*, the national health service surgical waiting list) based on patient-reported outcome measures	*Aliança Democrática*
Purchase of care from private providers	To issue specialist appointment vouchers, allowing patients free choice of provider when national health service waiting time targets are exceeded	*Aliança Democrática*
Expanded financial protection	Zero value added tax on menstrual hygiene products	*Partido Animais e Natureza*
Expanded coverage	Full drug coverage for individuals with income below the national minimum wage (in 2024: €11 480 annually)	*Bloco de Esquerda *
**Resource generation**		
Workforce planning, availability and distribution	To ensure maternal health professionals receive pregnancy, childbirth and postpartum human rights training provided by the specialty colleges of the nursing and medical societies	*Iniciativa Liberal*
	To guarantee fair remuneration and career progression for healthcare professionals by: revising salary grades; creating a system of individual or group incentives; reimbursing travel expenses for appointments more than 100 km away; recognizing the medical and nursing professions as high-risk and rapid burnout occupations	*CHEGA*
	To establish a health-care professional motivation plan to recognize the value of all human resources involved in health-care provision, especially in the national health service. This plan will comprise incentives, career development, flexible working hours, professional differentiation and new skill profiles	*Aliança Democrática*
	To offer incentives to the workforce in underserved areas: housing and family support, professional development, telemedicine, and integration into multidisciplinary teams	*Partido Socialista*
	To encourage full-time employment with non-compete agreements while working for the national health service by ensuring career progression and professional development, redirecting incentives towards this goal	*Partido Socialista*
	To establish full-time employment with non-compete agreements for physicians and nurses while working for the national health service	*Partido Animais e Natureza*
	To hire nutritionists for all national health service primary care units	*Partido Animais e Natureza*
	To establish full-time employment with non-compete agreements with a 40% higher salary and 50% additional career progression points	*Bloco de Esquerda*
	To reduce a physician's national health service minimum emergency care work week to 12 hours, freeing up time for appointments and surgeries	*Bloco de Esquerda *
Generation and the upkeep of infrastructure and medical equipment	To equip the larger national health service primary care units with laboratory and diagnostic tests procedures facilities	*Partido Socialista*
	To equip the larger national health service primary care units with laboratory and diagnostic tests procedures facilities, namely for radiographs and electrocardiograms	*Bloco de Esquerda *
	To promote the upkeep of national health service infrastructure by: assessing which hospital facilities require urgent renovation; investing in the construction of new buildings	*Livre*
**Service delivery**		
Care provision for a target population	To prioritize the assignment of family doctors to pregnant women, children younger than 9 years and adults older than 65 years	*Iniciativa Liberal*
	To expand HIV pre-exposure prophylaxis appointments to national health service primary care units and enable the dispensing of HIV-related drugs at community pharmacies	*Iniciativa Liberal*
	To implement chronic patient case management for frequent emergency department users	*Aliança Democrática*
	To deliver emergency and long-term care to elderly people through nurse teams that provide home visits and through home delivery of medication in coordination with local pharmacies	*Partido Socialista*
Emergency care	To strengthen gatekeeping for direct access to hospital emergency departments by generalizing telephone triage, which will steer users to either primary care or [other] hospital services	*Partido Socialista*
Public health	To conduct a nationwide nutritional quality and obesity risk study in every school and implement a nutritional and physical activity programme, promoted by the national health service primary care units in collaboration with schools and civil society	*Partido Animais e Natureza*
Mental health care	To focus on diversifying mental health responses and implementing a stepped care model; to promote the mental health organization model based on community multidisciplinary teams	*Livre*

€: euro; HIV: human immunodeficiency virus.

Note: These are the authors’ translations. The original text for the 350 classified measures is available in Portuguese upon request to the corresponding author.

Measures concerning the role of private providers, electronic health records, the governance of public hospitals and the national health services board, and health system performance monitoring were classified as governance, as they directly relate to strategic policy frameworks and health system regulation and oversight.

Financing concerns the flow of monetary resources through the health system (that is, raising and spending money on health care). Measures about pay-for-performance in either primary or hospital care were classified as financing as they relate to monetary incentives to prioritize the delivery of certain types of services while fostering quality. Measures about the purchase of care from private providers, expanded financial protection or expanded coverage were classified likewise.

Resource generation ensures that the health system has all the necessary inputs to produce health care. Therefore, measures concerning both the generation and the upkeep of infrastructure and medical equipment were classified as such. Measures concerning workforce planning, availability and distribution were also classified as resource generation.

Measures concerning emergency care, public health and mental health care, as well as those related to the provision of care for target populations, such as children, elderly people, human immunodeficiency virus (HIV)-positive patients and patients with chronic diseases, were classified as service delivery. According to the health systems assessment framework’s definition of service delivery, these measures include target populations; the measure’s primary purpose; type of provider and delivery platforms; and level and mode of service provision.

**Fig. 1:**
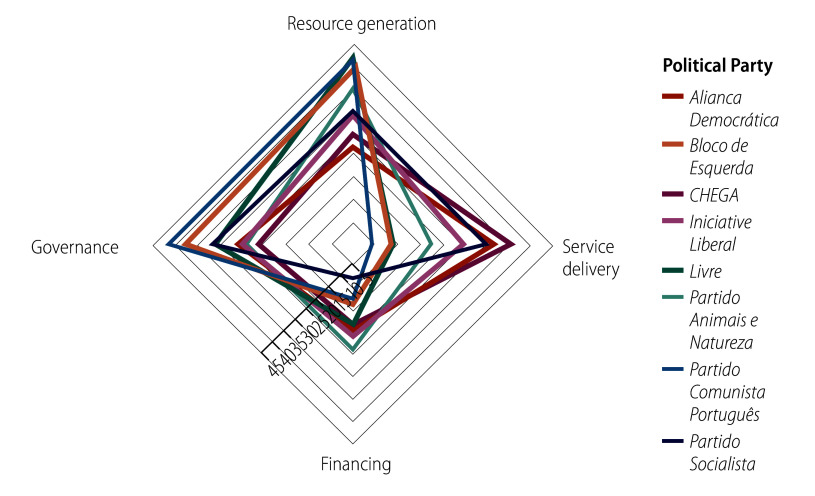
Analysis of percentage of measures by health system function and by political party, Portugal, 2024

Note: The number of measures per party is presented in Table 1.

Using the classifications as above, we compared how political parties weigh each function; three clusters of parties emerged (Fig. 1). *Partido Comunista Português*, *Bloco de Esquerda* and *Livre* (politically left-wing parties) focused on governance and resource generation. *Partido Animais e Natureza* and *Iniciativa Liberal* (whose self-declared political identity is not linked to the left/right spectrum) weighed functions relatively evenly. The *Partido Socialista* and *Aliança Democrática* (politically centrist parties which have alternately held office since 1975) devoted relatively more attention to service delivery, as did the politically far-right party *CHEGA*. *Partido Socialista*, *Aliança Democrática* and *CHEGA* differ to the extent that *Partido Socialista* attributes little absolute importance to financing and relatively more importance to governance than the other two.

## Lessons learnt

Authors have argued that there is a need for comparisons that “cluster and compare specific, policy-modifiable aspects of health systems – such as their governance, financing, the generation and deployment of resources, and the design of care delivery – aspects that are identified based on the policy question.”^5^ We have shown this argument also applies to the analysis of political parties’ manifestos within a country, allowing a comprehensive and structured synthesis of the parties’ health policy proposals. The lessons learnt from this exercise are summarized in Box 1. Note that our analysis is not intended to assess parties’ track records in delivering on these manifestos.

Box 1 **Summary of main lessons learnt**

- The health systems assessment framework can be used to synthesize and analyse political manifestos, enabling systematic comparisons of parties’ health policy vision and position, and used to identify clusters of parties.
- Expert knowledge of party rhetoric and political and institutional context are necessary to inform the classification process.
- The health systems assessment framework can reveal missing elements in the health policy debate; the challenge that remains is distinguishing between truly missing topics and those implicit in broader statements, so multiple rounds of review and cross-checking among experts in expert consensus meetings is key to reaching an outcome.

Classifying health policy measures by health system function is the stepping stone for further insights. First, the classification enables characterization of each party according to the weight given to each health system function. Second, it allows for cross-party comparisons. Political strategists may use this analysis to ensure that their party's manifesto covers all health system functions, and identify areas where their party's manifesto may be underdeveloped compared to other parties. Third, the approach enables the identification of clusters of parties.

The same authors also argued that the comparison of policy-modifiable aspects should be conducted based on particular policy questions. We show that, in the case of political manifestos, the comparison of policy-modifiable aspects of health systems should be conducted based on the full set of health policies included therein. This approach provides a comprehensive assessment and comparison of the party’s overarching vision for the health system, in contrast with the common approach to manifesto analysis, which has been to identify particular topics within them. For example, in July 2024, debate around the United Kingdom of Great Britain and Northern Ireland’s snap general election included debate on whether the political manifestos addressed women’s health, the elective care backlog or sugar taxes.^6^^–^^8^ However, such an issue-driven approach arguably offers voters, policy-makers and political strategists an incomplete assessment.^9^

One may ask whether this characterization is driven by the electoral timeframe – snap versus regularly-scheduled elections. Manifestos published ahead of a snap election may indeed differ from those published for an election at the end of a term. Yet, the difference is not straightforward. The long-term nature of health policy design means parties can generally draw from previous manifestos or government programmes when writing their new manifestos, potentially minimizing differences between snap and regular election proposals. On the other hand, the shortened timelines of snap elections might lead to greater focus on immediate issues rather than a long-term policy vision. Interestingly, the governing party's (*Partido Socialista*) manifesto largely reflected policies planned for their expected remaining term, which can reflect both the impact of rushed drafting due to short turnaround times and/or policies already in motion. Regardless, the classification process itself is robust to variation in electoral timeframes: while this variation might affect functions’ relative weights within manifestos, the classification of each measure depends uniquely on its content and the health systems assessment framework’s definitions.

Finally, the classified measures can also be analysed from a health systems function perspective, combining all political manifestos. This approach amounts to changing the unit of analysis from party manifestos to health system functions. For each function we checked whether the elements contained in the health systems assessment framework’s definitions, examples and sub-functions were reflected in the full set of 350 measures. This analysis allowed us to verify whether elements included in the health systems assessment framework were missing from the manifestos, and thus likely to be absent from the political debate. Most strikingly, we found that there were virtually no measures related to revenue raising within the financing function, which is broadly defined as spending and raising money on health care. This fact was captured and promptly broadcast to the public by the Portuguese media.^10^
